# Spectrum of Ocular Findings in Closed Head Injuries, Correlation With Severity of Neurological Involvement, and Treatment Outcome: A Hospital-Based Cross-Sectional Study

**DOI:** 10.7759/cureus.16515

**Published:** 2021-07-20

**Authors:** Shradha Pattnaik, Bijnya B Panda, Suresh C Swain

**Affiliations:** 1 Ophthalmology, Sriram Chandra Bhanja Medical College and Hospital, Cuttack, IND; 2 Ophthalmology, Sriram Chandra Bhanja Medical College, Cuttack, IND

**Keywords:** closed head injury, glasgow coma scale, relative afferent pupillary defect, traumatic optic neuropathy, ocular trauma

## Abstract

Purpose: To study the various ocular findings in patients with closed head injuries, to find any association with the degree of neurological involvement, and to analyze the treatment outcome after the necessary intervention.

Setting: Tertiary referral hospital in Eastern India.

Design: Prospective observational study.

Methods: Patients with closed head injuries attending our Outpatient department as well as referred from the Neurosurgery department for ophthalmic evaluation between October 2017 and September 2019 were recruited for the study. All patients meeting the inclusion criteria were examined by an experienced ophthalmologist. The Glasgow coma scale (GCS) was applied to grade the neurological involvement by the neurosurgery team. Ocular findings were recorded and necessary imaging was requested. Appropriate neurosurgery consultations were done in patients with neurological findings. All ocular injuries were managed as per institutional protocol. Descriptive statistics were used for analysis with p< 0.05 taken as statistically significant.

Results: A total of 207 patients (414 eyes) were included in the study. The mean age was 33.82 years, with the prevalence of male patients (82.12%). The most common cause of head injury was RTA (57.01%) followed by assault (11.59%). The majority of patients (53.14%) were classified as having moderate, 46.37% patients with mild, and 0.48% with severe neurological involvement as per GCS scoring. Isolated ocular findings were seen in 70.04% of patients while 29.95% of patients had both neurological and ophthalmic features. Ocular adnexal involvement was observed in 38.6%, anterior segment involvement in 86%, neuro-ophthalmic manifestations in 33.3%, and posterior segment involvement in 38.6% of patients. Ocular signs were resolved over due course of time in 48.8% of patients, completely resolved in 28%, while there was no improvement in 6.28% of patients. The final best-corrected visual acuity of >6/18 was achieved in 51.69% of patients. Statistical significance was observed in the correlation between the GCS scoring and general ocular findings (p= 0.02) as well as a relative afferent pupillary defect (p=0.003). The association between age > 50 years and neuro-ophthalmic features was not found to be statistically significant (p=0.56).

Conclusion: Poor visual acuity at presentation, optic canal fractures, the presence of multiple fractures of orbital walls, no improvement in vision within 48 hours of starting intravenous corticosteroids, were indicators of a poor visual prognosis in this study. The GCS, neuro-deficit, and ocular signs contribute significantly to the prediction of outcomes. Prompt treatment and referral can lead to a good resolution of symptoms and signs.

## Introduction

The overall incidence of traumatic brain injury globally is around 939 cases per 100,000 people and every year around 69.0 million people suffer from this “silent epidemic” [1]. India has unfortunately the highest rate of head injury in the world and it is also estimated that more than one million suffer from serious head injuries [2]. Closed head injury has been associated with ophthalmic findings with or without vision-threatening conditions in 25% -83% cases [3-6]. A higher incidence of ocular findings has been reported in studies where ophthalmologists take an active part during the evaluation of affected patients [4]. Triaging should be done at the screening stage to allow early medical and surgical intervention wherever indicated [7,8]. A multidisciplinary team approach is always required for a better prognosis of life and vision [9]. There are few studies that have tried to evaluate the association of ocular findings with the severity of head injury [4-6]. The present study was undertaken to describe the spectrum of ocular findings in patients with a closed head injury, correlate with the degree of neurological involvement and study the outcome of early management in such patients.

## Materials and methods

This prospective observational study was conducted in a tertiary hospital in Eastern India from October 2017 to September 2019 after approval from Institutional Ethics Committee (S C B Medical College, Cuttack, Odisha, India, approval number 988/14.10.2019) and adheres to the tenets of the Declaration of Helsinki. Data were collected after written informed consent from the patient and/or relatives. Consecutive sampling was done to avoid bias.

Participants

All adult patients of >18 years diagnosed with ocular trauma due to closed head injury attending ophthalmology OPD and those referred from the Neurosurgery department for evaluation were included in the study. Patients with follow-up <6 months, critically ill and unconscious patients, and those who did not give consent for the study were excluded from the study.

Data collection and study variables

After thorough history taking of patients or their close relatives, an ophthalmological examination was done. The evaluation was done in Out Patient Department if the patient was ambulatory or bedside examination was done when necessary. The Glasgow coma scale (GCS) was applied to grade the severity of the injury and assess the prognosis. The data regarding the demographic profile, mode of head trauma, length of time from injury to examination, type of trauma sustained, the extent of injuries (including neuro-imaging), loss of consciousness, and findings of neurological and ophthalmic evaluations were recorded. Ophthalmic examination included assessment of best-corrected visual acuity, pupillary response, extra orbital injury assessment, extraocular movements, anterior segment evaluation with a slit lamp, visual field assessment by confrontation/perimeter, intraocular pressure by Schiotz indentation tonometer/ non-contact tonometer, diplopia charting, and gonioscopy. CT scan, MRI, and B-Scan were done when indicated. Patients were followed up after one week, four weeks, and 24 weeks. The head injury was graded as follows: mild head injury: GCS 13-15, moderate head injury: GCS 9-12, and severe head injury: GCS 3-8.

Study size

Five hundred forty patients were recruited for the study. Two hundred seven patients met the inclusion criteria and were included in the study for analysis. We excluded 47 patients who died during the early course of follow-up due to severe neurological complications.

Statistical analysis

The data collected were analyzed statistically using descriptive statistics and depicted in the form of like frequency and percentages. The “chi-square” test was used to analyze categorical data, to find any association between the GCS and manifestations of closed head injuries. P<0.05 was considered statistically significant.

## Results

In this study of 207 patients of ocular trauma due to closed head injury, 170 were males (82.12%) and 37 were females (17.87%). The age was 18 to 67 years (mean 33.82 years). The most common age group affected was 21-30 years.

Road traffic accidents were the most common cause of head injury, accounting for 55.07% followed by assault 11.59%, others being due to occupational hazards, domestic violence, fall from height, and sports injuries. The major causes of head injury recorded in the study have been illustrated in Table 1.

**Table 1:** Major causes of head injury RTA - Road traffic accident

Cause	Number	Percentage
RTA	114	55.07%
Assault	24	11.59%
Fall from height	07	3.38%
Domestic injury	16	7.72%
Occupational	16	7.72%
Hitting head with wall	05	2.41%
Fall of objects on the head	08	3.86%
Sports injury	07	3.38%
Blast injury	07	3.38%
Animal attack	02	0.96%
Gunshot	01	0.48%

Isolated ocular involvement was seen in 70% (n=145) and 30% (n=62) patients had both ophthalmic and neurological involvement.

Best corrected visual acuity >6/18 was recorded in 63.2% (n=262 eyes), 18% (n=75 eyes) had BCVA > 6/60 and 18.8% (n=77 eyes) had BCVA < 5/60.

The commonest eye signs were lid edema (14%) followed by peri-orbital ecchymosis in 10.6%. Subconjunctival hemorrhage was the commonest soft tissue manifestation, i.e., 58 of 207 patients (28%). Eighteen patients (8.6%) suffered some type of orbital fracture and the lateral orbital wall was most commonly fractured in seven cases (3.38%). The most common intraocular injury was hyphema seen in 19 cases (9.17%), traumatic cataract in 11 cases (5.31%), vitreous hemorrhage in 11 patients (5.31%), retinal hemorrhage in 20 patients (9.66%), macular edema 13 patients (6.28%) and traumatic optic neuropathy (TON) in 10 patients (4.83%). The detailed ocular findings have been illustrated in Table 2.

**Table 2:** Ocular findings in patients classified according to their severity of neurological involvement TON - Traumatic optic neuropathy

GCS score	No. of patients	OCULAR FINDINGS	PERCENTAGE
<9 (severe)	1	TON (1)	100%
9-12 (moderate)	110	LID TRAUMA (7)	6.36%
		6^TH^ NERVE PALSY (8)	7.27%
		3^RD^ NERVE PALSY (6)	5.45%
		4^TH^ NERVE PALSY (5)	4.54%
		TON (8)	7.27%
		MACULAR EDEMA (13)	11.81%
		PAPILLEDEMA (4)	3.63%
		MULTIPLE CRANIAL NERVE PALSY (3)	2.72%
		FIELD DEFECT (5)	4.54%
		BLUNT GLOBE TRAUMA (1)	0.90%
		TRAUMATIC MYDRIASIS (11)	10%
		OPTIC ATROPHY (1)	0.90%
		ORBITAL FRACTURES (11)	10%
		RETINAL HEMORRHAGE (9)	8.18%
		AC HYPHEMA (2)	1.81%
		DISLOCATION/SUBLUXATION OF LENS (1)	0.90%
		ORBITAL HEMATOMA (2)	1.81%
		BASAL SKULL FRACTURE (2)	1.81%
		COMMOTIO RETINA (4)	3.63%
		MACULAR HOLE (3)	2.72%
		CHOROIDAL DETACHMENT/RUPTURE (1)	0.90%
		INTERNAL OPHTHALMOPLEGIA (1)	0.90%
		CORNEAL FOREIGN BODIES (3)	2.72%
		EMPHYSEMA ORBIT (1)	0.90%
		NYSTAGMUS (1)	0.90%
13-15	96	BROW TEAR (1)	1.04%
		LID TRAUMA (16)	16.66%
		6^TH^ NERVE PALSY (7)	7.29%
		TRAUMATIC CATARACT (11)	11.45%
		3^RD^ NERVE PALSY (7)	7.29%
		TON (1)	1.04%
		PAPILLEDEMA (4)	4.16%
		ORBITAL FRACTURE (1)	1.04%
		IRIDOCYCLITIS (3)	3.12%
		ANTERIOR CHAMBER HYPHEMA (18)	18.75%
		DISLOCATION/SUBLUXATION OF LENS (6)	6.25%
		CORNEAL ABRASIONS (6)	6.25%

In our study, the commonest cranial nerve to be affected was the abducens nerve followed by the oculomotor nerve. Extraocular movement restriction was present in 31 patients (14.97%). Abnormalities in the pupillary reaction were found in 27.53% (57 patients) with random amplification of polymorphic DNA (RAPD) seen in 12 patients (5.79%).

The correlation of ocular findings with the GCS in our patients showed that 46.37% of patients (n=96) had a mild head injury (GCS 13-15) and 53.14% of patients (n=110) had a moderate head injury (GCS 9-10). In patients with a moderate degree of head injury (n=110), 43 patients had neurological features and 67 patients had ocular features in contrast to mild head injury patients (n=96), whereas 23 patients had neurological features and 73 patients had ocular features.

The association of EOM restriction and cranial nerve palsies was found to be non-significant, p-value > 0.05. The association of RAPD grades with poor visual outcomes was found to be significant in 12 patients (p=0.003). After treatment at six months, follow-up ocular signs were resolved over due course of time in 48.8% of patients, completely resolved in 28%, while there was no improvement in 6.28% of patients. The final best-corrected visual acuity >6/18 was achieved in 51.69% of patients. Statistical significance was observed in the correlation between the GCS scoring and general ocular findings (p= 0.02) as well as a relative afferent pupillary defect (p=0.003). The association between age > 50 years and neuro-ophthalmic features was not found to be statistically significant (p=0.56).

## Discussion

A comprehensive ophthalmic evaluation is mandatory in all closed head injury patients for better prognosticating the visual outcome. In the present study, we have tried to study the spectrum of ocular findings, their association with the degree of neurological involvement, and their treatment outcome in such patients.

In the present study, the most common age group affected were in the range of 21 to 30 years with male predominance similar to other studies. Young people were more affected probably due to the increased association with outdoor activities, their risk-taking behavior, and alcohol abuse. Road traffic accidents were the most common cause of head injury (55.07%) followed by assault (11.59%). Most of the studies done in developed as well as developing countries have found similar results. In our study, BCVA > 6/18 was found in 63.2% (n=262 eyes), 18% (n=75 eyes) had BCVA > 6/60 and 18.8% (n=77 eyes) had BCVA < 5/60. These findings are similar to the study by Brahm et al. who found that of the inpatients (with moderate to severe head injury), 77.8% had BCVA > 6/18, 12.7% had <6/36, and 3.2% had no light perception in both eyes. Of the outpatients (with a mild head injury), 98.4% had a visual acuity of >6/18, 1.6% had a visual acuity worse than 6/36, which suggests that the majority of patients in both inpatients and outpatients had mild vision loss similar to our study [10].

Prevalence of ocular involvement in our study was 100% (70% isolated ocular involvement and neuro-ophthalmic involvement in 30%), while Kulkarni et al. and Sharma et al. have reported a prevalence of 83.5% and 49.7%, respectively, in their case series. Similar results are reported by Raju who has shown 100% ocular involvement [11]. These variable percentages may be due to the difference in the patient population. The commonest eyelid findings were eyelid edema (14%) followed by peri-orbital ecchymosis (10.6%). Subconjunctival hemorrhage was the commonest soft tissue manifestation seen in 58 of 207 patients (28%). Similar results were reported by Raju who has shown ecchymosis of the lids and sub-conjunctival hemorrhage in 30% of cases.

We have classified the ocular findings according to the severity of neurological involvement. In mild head injury cases with GCS 13-15 (n=96), the most common ocular findings were limited to anterior segment involvement including hyphema (18.75%) followed by eyelid laceration (16.66%) and traumatic cataract (11.45%). Our findings correlate with Kulkarni et al. where ocular involvement was seen in 82.7 patients and not of neurological importance and confined to extraocular manifestations, hyphema, and vitreous hemorrhage.

In patients classified as having moderate severity of neurological involvement with GCS 9-12 (n=110), the cause of vision loss was mostly due to macular edema (11.8%) and traumatic mydriasis (10%), and orbital fractures (10%).

There was a significant association (p=0.02) between ocular findings and severity of neurological involvement similar to other studies done by Kulkarni et al. and Sharma et al. The association of EOM restriction and cranial nerve palsy was found to be non-significant (p=0.07) suggestive of other causes of restricted ocular movements due to mechanical causes and inability to follow commands in severe head injury.

Abnormalities in the pupillary reaction were found in 27.53 % (57 patients) cases in our study. Poor visual outcomes had a significant association with the presence of RAPD in our patients. In the cases studied by Raju, pupillary abnormalities were present in about 55% of patients. Pupillary abnormalities were found in 7.95% as in a study by Kulkarni et al. in another study by Sharma et al. encountered pupillary abnormalities in 21.4% of cases. Faith et al. reported pupillary findings in 21.7% of cases [12].

In our study, 10 patients with TON who were administered intravenous methylprednisolone (IVMP) showed variable improvement in vision. Our results were similar to Bhattacharjee et al. who have reported that in their retrospective study including 18/35 cases of indirect TON who received IVMP showed similar visual improvement as the other group, which did not receive IVMP [13]. A study done by Rajiniganth et al. showed an overall improvement of 48% where they combined IVMP along with endoscopic optic nerve decompression [14].

There are certain limitations to our study. The sample size of our study population is small, which needs to be validated in a larger cohort of patients. We have excluded patients who succumbed during the study which might have affected few outcome measures. We could not use visual evoked potential in the assessment of optic nerve involvement in the acute setting, which might have resulted in missing few patients with TON.

## Conclusions

Early assessment of ocular signs and GCS scoring of neurological involvement can contribute significantly to the prediction of outcomes as evident from the statistical validity results in our study. Patients with ocular signs and neuro-deficit exhibited a progressively worse outcome as the GCS scoring worsened. Pupil size and reaction are important in the initial assessment of head injury, which aids in localizing the site of injuries as supratentorial, extradural, subdural, and pontine involvement. The presence of poor visual acuity at presentation, optic canal fractures, multiple orbital wall fractures, and no improvement in vision after administration of intravenous steroids are indicators of poor visual prognosis. A multidisciplinary team approach including ophthalmologist, neurosurgeon, and otolaryngologist is essential in the proper management of these patients.
